# Genes associated with chloroplasts and hormone-signaling, and transcription factors other than *CBF*s are associated with differential survival after low temperature treatments of *Camelina sativa* biotypes

**DOI:** 10.1371/journal.pone.0217692

**Published:** 2019-05-31

**Authors:** David Horvath, James V. Anderson, Wun S. Chao, Puying Zheng, Miles Buchwaldt, Isobel A. P. Parkin, Kevin Dorn

**Affiliations:** 1 USDA/ARS, Edward T. Schafer Agricultural Research Center, Sunflower and Plant Biology Research Unit, Fargo, North Dakota, United States of America; 2 Department of Plant Science, North Dakota State University, Dept., Fargo, North Dakota, United States of America; 3 Agriculture and Agri-Food Canada, SK, Canada; 4 Department of Plant Pathology, Kansas State University, Manhattan, Kansas, United States of America; Iwate University, JAPAN

## Abstract

Winter annual biotypes of *Camelina sativa* regularly survive after winter conditions experienced in northern regions of the U.S., whereas summer annual biotypes do not. To determine potential molecular mechanisms associated with these biotype differences in survival after low temperature treatments, we examined genetic and transcript variations in both a winter- (Joelle) and a summer- (CO46) biotype. It was determined that as few as one or two dominant genes may control differential survival after low temperature treatments. Of the 1797 genes that were differentially expressed in response to cold in both the winter and summer biotypes many COR genes were identified, indicating that the CBF regulon is functional in both. However, only 153 and 76 genes from Joelle and CO46, respectively, were either differentially expressed or not expressed at all in one biotype versus the other following cold acclimation. We hypothesize that these 229 genes play a significant role in, or are primarily responsive to, differences in survival after freezing between these two biotypes. Promoter analysis provided few clues as to the regulation or these genes; however, genes that were down-regulated specifically in the winter biotype Joelle were enriched with the sequence TGGCCCTCGCTCAC, which is over-represented among genes associated with chloroplasts in Arabidopsis. Additionally, several genes involved in auxin signaling were down-regulated specifically in Joelle. A transcription factor with strong similarity to *MYB47*, known to be up-regulated by salt, drought, and jasmonic acid, but not cold in Arabidopsis, was essentially off in the freezing sensitive biotype CO46, but was cold-induced in the winter biotype Joelle. Several other transcription factors genes including three with similarity to *WRKY70*, that may be involved in SA/JA-dependent responses, a *HOMEOBOX 6* gene involved in ABA signaling, and two others (*NUCLEAR FACTOR Y* and *CONSTANS-like 2*) known to be implicated in photoperiodic flowering were also differentially expressed between the two biotypes.

## Introduction

Camelina [*Camelina sativa* (L.) Crantz] is an oilseed crop that is composed of both summer- and winter-annual biotypes [1]. The oil produced from the seed of Camelina is valued by industry for production of biofuels, heart-healthy edible oils, and bio-based pharmaceuticals, and the crushed seed meal has been approved as a feed supplement for chickens and cattle [1,2]. Additionally, the winter-hardy traits of winter annual biotypes of Camelina allow them to be used as oilseed cover crops in the colder northern climates of the United States. As such, they provide ecosystems services including reducing soil erosion, nutrient retention, and early season-weed suppression and -nutrition for pollinators [2]. Winter annual biotypes of Camelina with high rates of survival following freezing conditions and early maturity traits allow for agricultural intensification through development of economically sustainable relay- and double-cropping systems in the upper Midwest and northern Great Plains [3].

Camelina and the model plant Arabidopsis (*Arabidopsis thaliana* L.) are both members of the Brassicaceae lineage I Camelineae tribe (4). Although the reference genome of *C*. *sativa* shares similarity to the Arabidopsis genome, Camelina genomics are more complex due to its hexaploid nature. Often, three paralagous copies of most Arabidopsis genes are present in the Camelina genome. However, based on the high degree of synteny and functionality observed between annotated genes of Camelina and Arabidopsis, their gene products are proposed to play similar roles in biological pathways and processes [2,4].

Survival after freezing is an agriculturally important trait for winter cover crops [5]. Winter biotypes of Camelina, such as Joelle, must accumulate adequate cold acclimation to withstand the extreme freezing conditions of the northern Great Plains of the U.S. To survive freezing conditions that might occur from time to time in the early fall or late spring, winter biotypes must also be able to rapidly acclimate or reacclimate. In the case of intermittent late spring freezing conditions, the same is true for spring biotypes, such as CO46. In our studies, the winter biotype Joelle has been observed to have significantly greater survival rates following exposure to freezing temperatures than the summer biotype CO46.

Cold acclimation and freezing survival processes are well-studied phenomena at the genetic and physiological levels. Several key regulators of the cold acclimation response, such as the CBF regulon, have proven to be sufficient for improving freezing survival [6] and are activated following exposure to low but non-freezing temperatures [7]. However, many plants which are incapable of significant cold hardening appear to have functional CBF genes and proteins that are also cold-induced [8–10]. Thus, there is some question as to what differences result in enhanced freezing survival between species or even between different cultivars within species that appear to possess active cold-acclimation signaling. Thus, the winter and summer biotypes of Camelina, along with their extensive genomics tools [1,2,4,11], provide a unique model system to examine genetic and molecular mechanisms associated with cold acclimation responses leading to survival following exposure to freezing conditions.

In this study, we analyze RNAseq and whole genome sequence to identify key molecular components of the cold acclimation process in winter- and summer-biotypes of Camelina with divergent survival after freezing highlighted gene regulatory networks and *cis-acting* elements of coordinately-expressed gene clusters. By examining the genetics and differential expression of genes that distinguishes survival after low temperature treatments between Joelle and CO46, our objective was to identify potential targets for improving survival after freezing in winter annual brassica species such as Camelina and canola (*Brassica napus*).

## Materials and methods

### Plant growth, cold acclimation, and low temperature treatment

The methods for plant growth and conditions for cold-acclimation treatment of winter- and summer-annual biotypes of Camelina described here are the same as previously described by (1). Briefly, seeds were planted in Deepot Cells (D60L: 6.4 cm X 35.6 cm, 983 ml volume; Stuewe &Sons, Inc., Tangent, OR, USA) contaning potting soil (Sun-shine mix #1; Fisons Horticulture Inc., Bellevue, WA, USA), and grown under greenhouse conditions for 4 weeks with daily watering and weekly fertilization. Plants were then placed in temperature-controlled walk-in growth chambers at 5°C under 12 h photoperiods augmented with full spectrum LED lighting with an average intensity of 4500 lux at plant level (Lumibar pro, Lumigrow Inc. 1480 64th Street, Suite 150, Emeryville, 94608, California, USA) for 8 weeks prior to freezing. Watering was done as needed during acclimation (generally once every two-three weeks). Low temperature treatments were accomplished by subjecting plants to an 12 h linear ramp down from 22°C to -15°C, holding at a constant -15°C for 4 h, followed by an 8 h ramp up to 22°C. Plants were then moved to the greenhouse for damage scoring and survival assessment. All freezing runs were initiated at 8:00 AM with the normal 12 h photoperiod. No nucleating agents were added to allow undercooling to occur if that was an active mechanism impacting the stark survival differences between the two acclimated biotypes. Following the freezing treatment, plants were returned to ambient greenhouse conditions for evaluation over 7 days.

### Crosses and genetic analyses

Winter biotype (Joelle) plants were grown to the six-leaf stage under greenhouse conditions (10 h photoperiod with temperatures of ~22–26°C). These plants were then acclimated for 8 weeks at 5°C under 12 h photoperiods in temperature-controlled walk-in growth chambers. Approximately 4 wks before the end of vernalization, summer biotype (CO46) plants were started under greenhouse conditions to synchronize flowering of the summer- and winter-biotypes. Reciprocal crosses between the two biotypes were made, and the resulting F_1_ seeds were planted. The resulting seedlings from F_1_ seed were grown under greenhouse conditions as previously described. Five confirmed F_1_ seedlings from 8 sets of reciprocal crosses were tested for their survival after low temperature treatments as described above. Since both reciprocal crosses demonstrated similar survival after freezing, one line was selected and self-fertilized to generate an F_2_ population. Seeds from this population were grown under greenhouse conditions and acclimated/vernalized for 8 weeks and then subjected to freezing as described above. The damage resulting from the freezing treatment was assessed at two weeks after treatment on a 0 to 3 scale as follows: 0 = dead, 1 = more than 70% leaf damage but surviving merisemtatic tissue, 2 = between 30% and 70% leaf damage and surviving meristematic tissue, 3 = between 30% and 0% leaf damage and surviving meristematic tissue. A Chi square analysis was done to determine the most likely inheritance mechanisms associated with survival. Remaining seeds (254) from the F2 population were grown to establish a RIL population that was advanced by single seed descent for 5 additional generations. One individual from each F5 RIL family was subsequently phenotyped for damage scores as described above. Additionally, the freezing treatment-induced damage to the photosynthetic apparatus of Camelina plants was analyzed by measuring the dark-adapted chlorophyll fluorescence (Fv/Fo and Fv/Fm). This was accomplished using an OS30p+ hand held chlorophyll fluorometer (Opti-Sciences, Inc., 8 Winn Ave., Hudson NH), which provides a measure of photosystem II activity and is a very sensitive stress detector.

### RNAseq, whole genome sequencing, and bioinformatic analyses

Shoot tips including young leaf and meristem material were collected from plants either immeadiately prior to beginning (unacclimated), or at the end of the 8 week cold treatment (acclimated) described above. Plant material for all treatments were collected between 10:00 AM and 2:00 PM to avoid diurnal differences from being detected. RNA was extracted as described [1], as was RNA isolated from the winter- (Joelle) and summer- (CO46) annual biotypes for RNAseq and bioinformatic analyses. Briefly, only annotated transcripts with Fragments Per Kilobase of transcript per Million (FPKM) >5; False Discovery Rate (FDR) <0.05; and Differential Expression (DE) >2X were included in our analyses. Sequence data was deposited into NCBI BioProject ID = PRJNA292793 and trimmed and filtered RNAseq reads were mapped to the reference genome of Camelina (4 – http://www.Camelinadb.ca/downloads.html). Promoter sequences (1500 bases 5′ to the putative translation start site) were obtained using BEDTools v2.15.0 [12] from the spring line DH55, which has served as the reference sequence for Camelina [4]. These 1500 bp regions were intersected with the negative gene space to eliminate overlaps with neighbouring genes. In cases where genes were wholly contained within the putative promoter region, a Perl script was written to identify the available segment adjacent to its associated gene and filter out the disconnected regions. The resulting promoter fasta files from subsets of genes (genes that were either up, down, or off and specific to Joelle or CO46) were searched for over-represented sequences using the web-based program MEME, and any sequences that were identified were used to search for over-represented gene ontology categories using the web-based program GOMo [13].

## Results

### Genetic analysis of survival after low temperature treatments between Joelle and CO46

**Fig 1:**
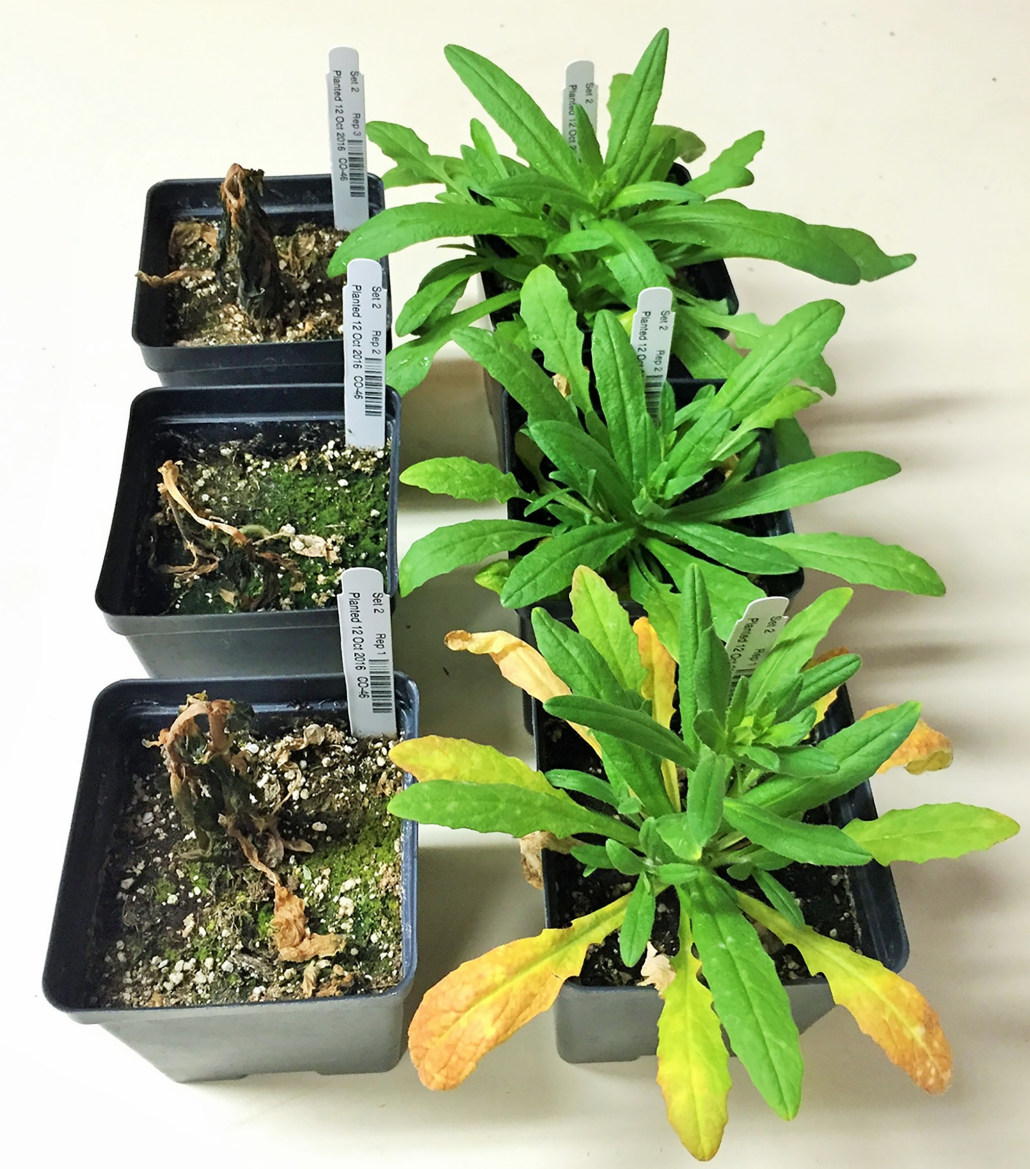
Photos of freezing damage in parental biotypes. Photo of three individual freezing sensitive biotype CO46 (left) and winter biotype Joelle (right) following 8 wks at acclimating conditions, subsequent freezing at -15°C for 4 hrs, and recovery in the greenhouse for 2 wks.

**Fig 2:**
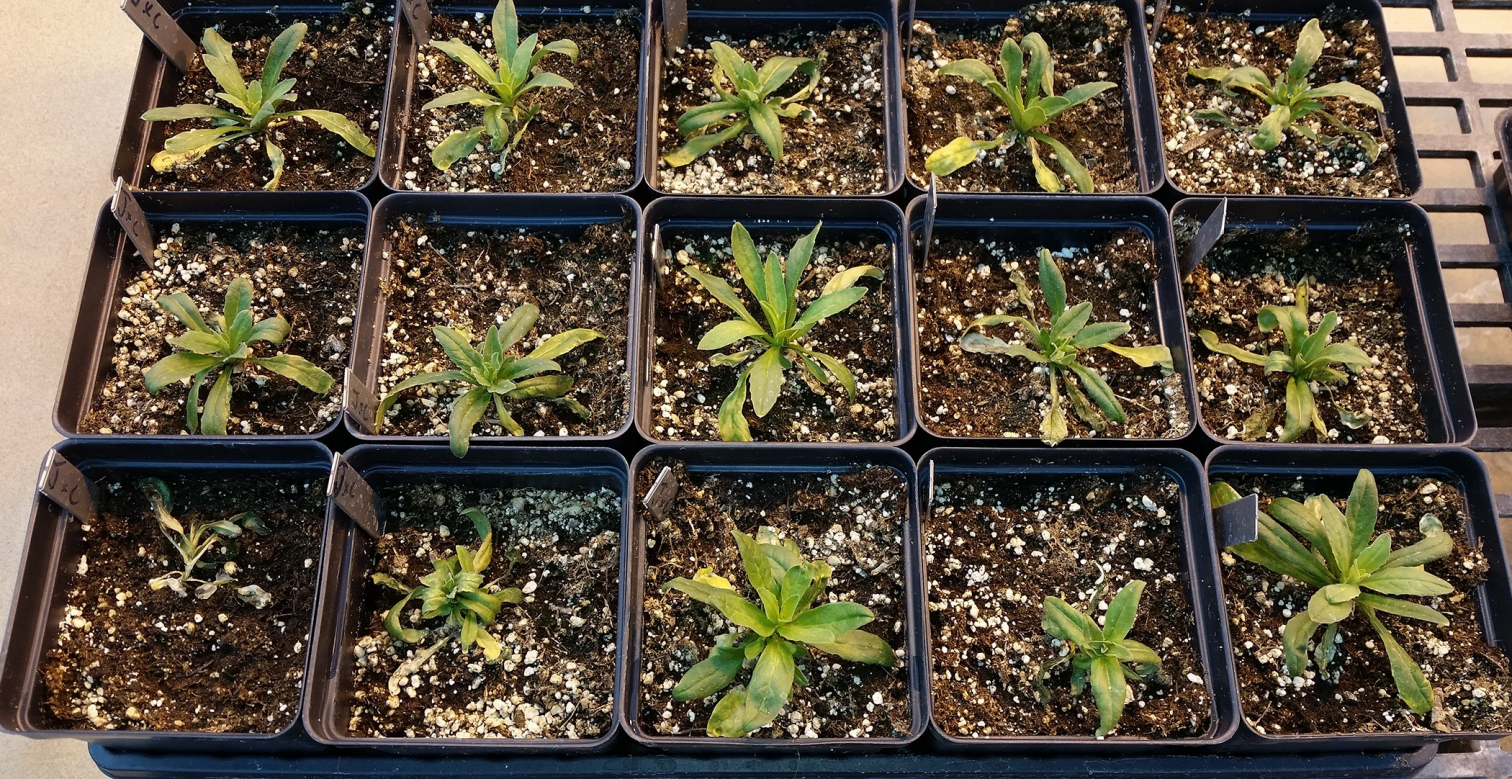
Photos and freezing damage scoring of F2 plants. Photo of a representative set of 15 F2 Camelina plants after 8 wks acclimation, subsequent freezing at -15°C for 4 hrs, and recovery for 1 week in the greenhouse. Scoring for this set (0 = dead, 3 = minimal or no damage) from left to right and top to bottom was as follows: 3,3,3,3,2,3,3,3,2,1,0,1,3,2,3.

The summer biotype CO46 and the winter biotype Joelle have differences in their ability to cold harden and survive subsequent freezing (Fig 1). Crosses made between these two biotypes indicated that survival after freezing appears to be primarily regulated by one or more dominant genes. Most of the F_1_ progeny tested, regardless of the reciprocal state of the cross, demonstrated similar survival after low temperature treatments as did the Joelle parent (S1 Table). F_2_ plants generated from the selected cross appeared to segregate for survival after low temperature treatments, however some individuals had intermediate phenotypes (Fig 2). Although most of the progeny were observed to have freezing survival similar to the Joelle parent, about 1/3 had partial to significant freezing damage, and ~10% were killed by the freezing stress (Table 1).

**Table 1:** Chi square analysis from 30 F2 seedlings.

**score (damage)**	**3**	**2**	**1**	**0**	
**Observed**	15	7	5	3	
**Expected**	16.875	5.625	5.625	1.875	**accept (0.73)**

Chi square analysis data from 30 F2 seedlings. For the following hypotheses: Two dominant genes sorting independently with a 9:3:3:1 ratio where a dominant allele in either would protect the offspring from death, but where one gene had a greater impact on reducing damage than the other.

All Joelle parental controls had minimal to no freezing damage, and all CO46 parental controls were killed by the freezing treatment. The simplest explanation, supported by a Chi square analysis with an acceptable p value of 0.73, suggests survival after low temperature treatments is controlled by two dominant genes where one copy would be sufficient to provide partial protection (Table 1). However, we cannot rule out a more complex genetic control with this test.

**Fig 3:**
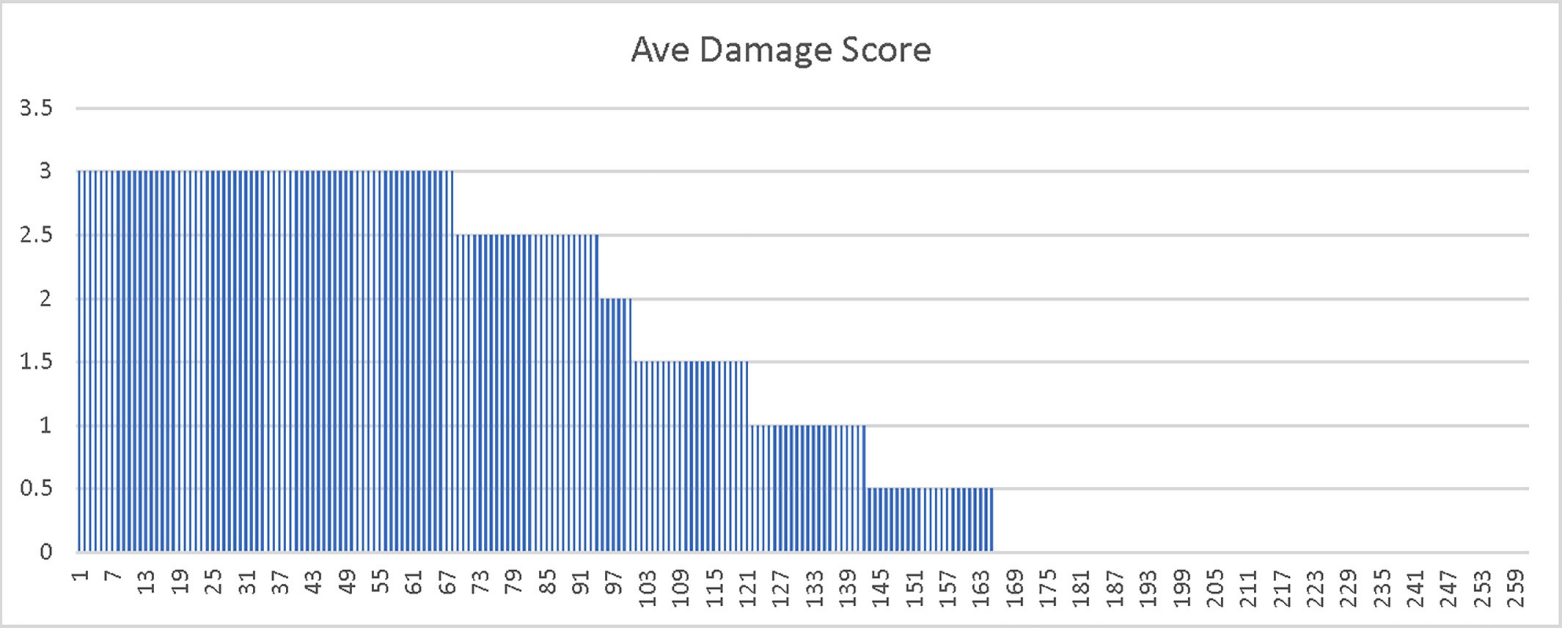
Damage scores from RIL families after low temperature stress. Damage score (see Fig 2 for a photographic representation of the 0–3 damage score ratings described in the methods section) representing an individual from each of 254 F2:F5 RIL families two weeks following low temperature treatments described in the methods section. The Y axis is the average damage score obtained from two independent ratings of the population.

**Fig 4:**
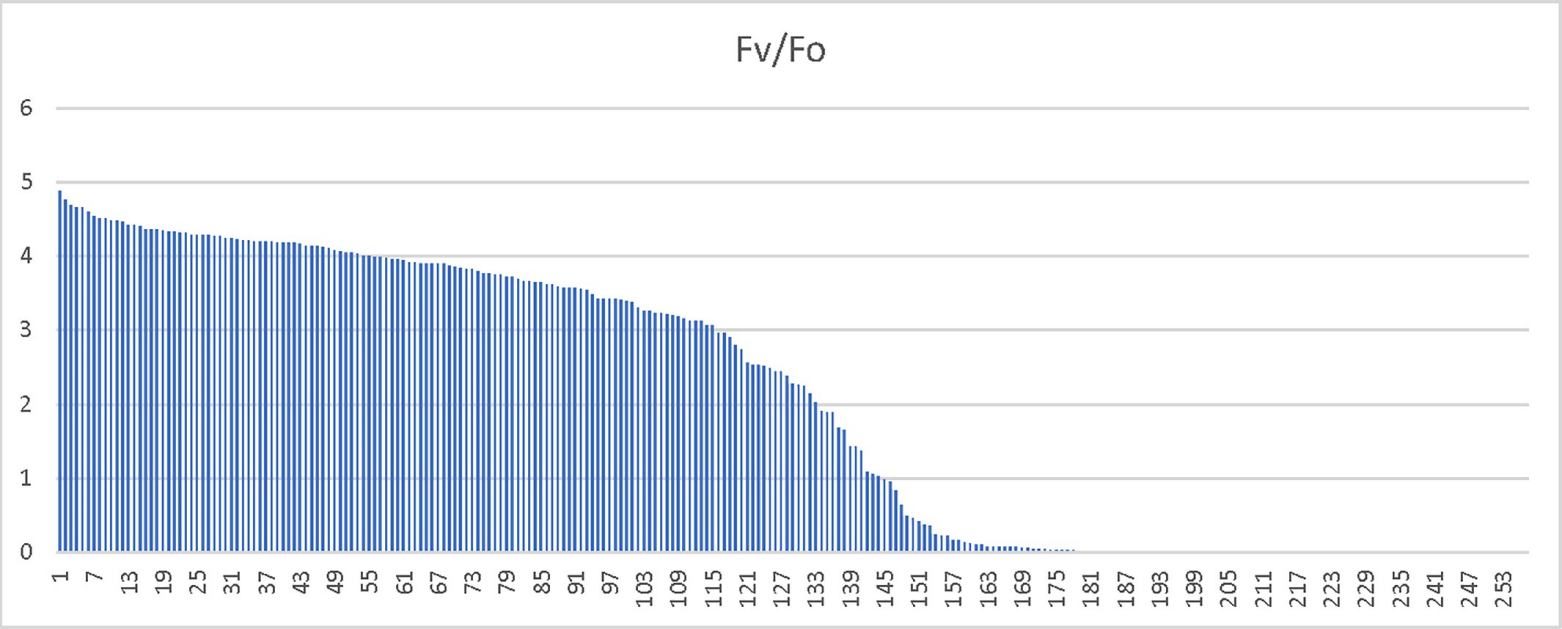
Chlorophyll fluorescence values from RIL families after low temperature stress. Chlorophyll fluorescence measures (Fv/Fo) for an individual from each of 254 F2:F5 RIL families two week following low temperature treatments described in the methods section. The Y axis represents the Fv/Fo values which are indicative of physiological stress.

**Fig 5:**
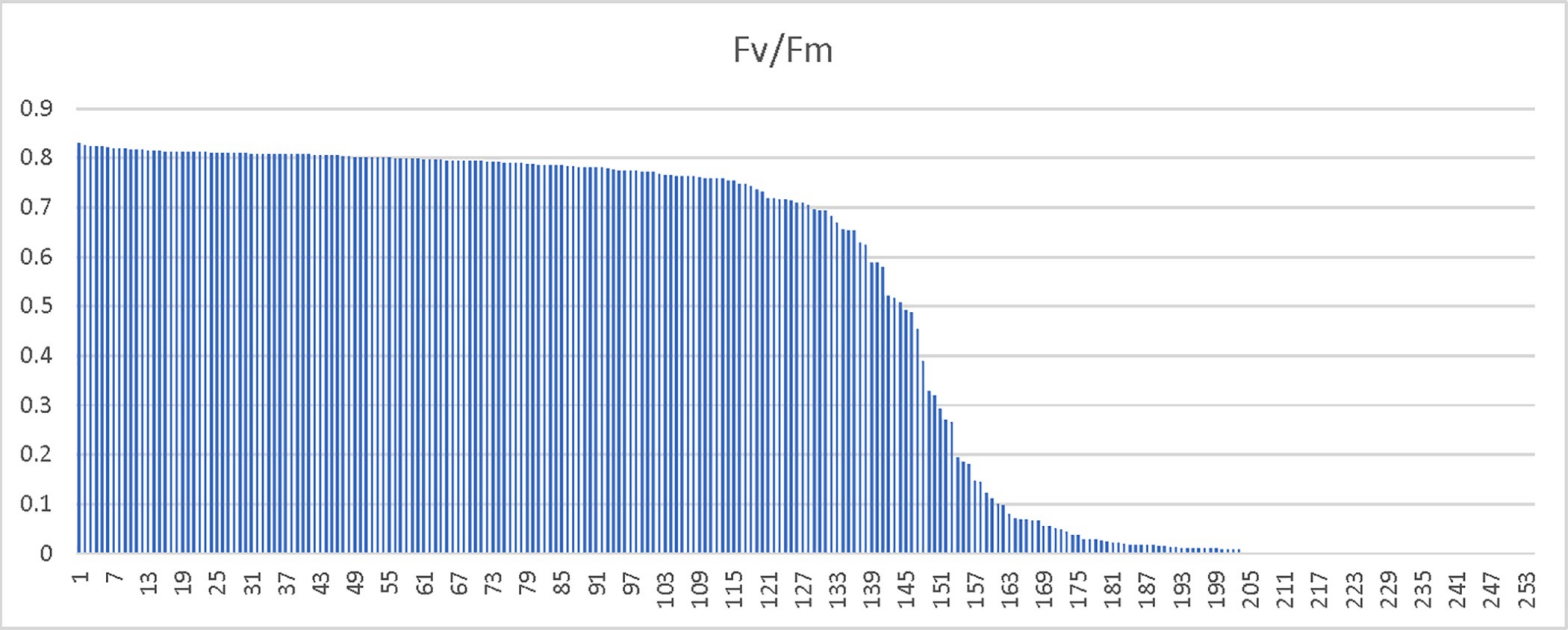
Chlorophyll fluorescence values from RIL families after low temperature stress. Chlorophyll fluorescence measures (Fv/Fm) for an individual from each of 254 F2:F5 RIL families two week following low temperature treatments described in the methods section. The Y axis represents the Fv/Fm values which are also indicative of physiological stress.

To further investigate the genetics underlying the differences in freezing tolerance between these two biotypes, we advanced 254 RILs by single seed decent- a process that should result in plants that are homozygous in a 1:1 ratio for all segregating alleles in the original cross. If the trait was highly polygenic, with alleles providing small incremental impacts on freezing tolerance, one would expect a relatively smooth range of phenotypes across the RIL families. However, if there were just two genes, one should expect to see four distinct groups, and if only a single major gene controlled the difference in freezing survival between these two biotypes, only phenotypes identical to the parents in regards to freezing survival would be expected. The results below are closest to what is expected from a single major gene controlling freezing survival (Fig 3). Because there are too many plants with a 0 rating (110 observed with an expected of 63.5), too many with little to no apparent damage (94 observed with an expected of 63.5) and too few plants had intermediate scores, the ratio does not pass a Chi Square test for a 1:1:1:1 ratio. Also, because a discontinuous scoring scale can skew interpretation of results by arbitrarily binning individuals into a small number of classes, we also examined chlorophyll florescence (Fv/Fo and Fv/Fm) as a measure of damage (Fig 4 and Fig 5 respectively). The Fv/Fo and Fv/Fm values, which are very sensitive detectors of stress, more clearly indicated two major clusters of about equal number, with a few intermediate phenotypes. Although these data are consistent with a small number of genetic differences influencing freezing survival differences in the two biotypes, it more closely supports a single major gene impacting the phenotype with possibly a number of genes that have only minor influences on the phenotypes of individuals from the original cross.

### Transcriptome differences between Joelle and CO46 after cold acclimation

Based on the criteria (FPKM >5; FDR <0.05; DE >2X), 2626 and 3406 genes were differentially expressed in response to cold acclimation within CO46 and Joelle, respectively (Table 2). Of those, 1797 (see Table 2; 983+796+11+7) were differentially expressed in response to cold acclimation in both biotypes. However, 18 of those had opposite trends in expression between biotypes; 7 genes were up-regulated in Joelle and down-regulated in CO46, and 11 genes were down-regulated in Joelle and up-regulated in CO46. Additionally, 829 and 1609 were unique to CO46 and Joelle, respectively. Further, analysis of transcriptome data identified 961 genes (see Table 2: Differential post acclimation) that were differentially expressed between the Joelle and CO46 biotypes following cold acclimation (comparing cold acclimated Joelle to cold acclimated CO46). Among these 961 transcripts, 192 are cold-regulated in both biotypes–obviously to different levels. Expression data for all annotated Camelina transcripts along with associated annotations can be found in S2 Table.

**Table 2:** Differentially expressed genes for indicated comparisons.

*Comparisons*	Joelle UP	CO46 UP	Joelle DOWN	CO46 DOWN
*Differential in Joelle and CO46 acclimated vs unacclimated*	1745	1405	1661	1221
*Differential post acclimation -Joelle vs CO46*	451	510	510	451
*Differential pre acclimation -Joelle vs CO46*	389	13	13	389
*Up in both -pre vs post acclimation*	983	983		
*Down in both -pre vs post acclimation*			796	796
*Biotype-specific up -pre vs post acclimation*	755	411		
*Biotype-specific down -pre vs post acclimation*			854	418
*Up in Joelle and down in CO46 -pre vs post acclimation*	7			7
*Up in Joelle and down in CO46- pre vs post acclimation*		11	11	

Cluster analysis of gene expression data in winter (Joelle) and summer (CO46) annual biotypes of *C*. *sativa*. Differential expression and trend is defined as FPKM >5; FDR <0.05; DE >2X. UP/DOWN refers to genes up- or down-regulated in the comparison noted in the first column, while the biotype for the comparision is noted in the column headers.

### Transcripts associated with cold acclimation treatments in winter biotypes

**Fig 6:**
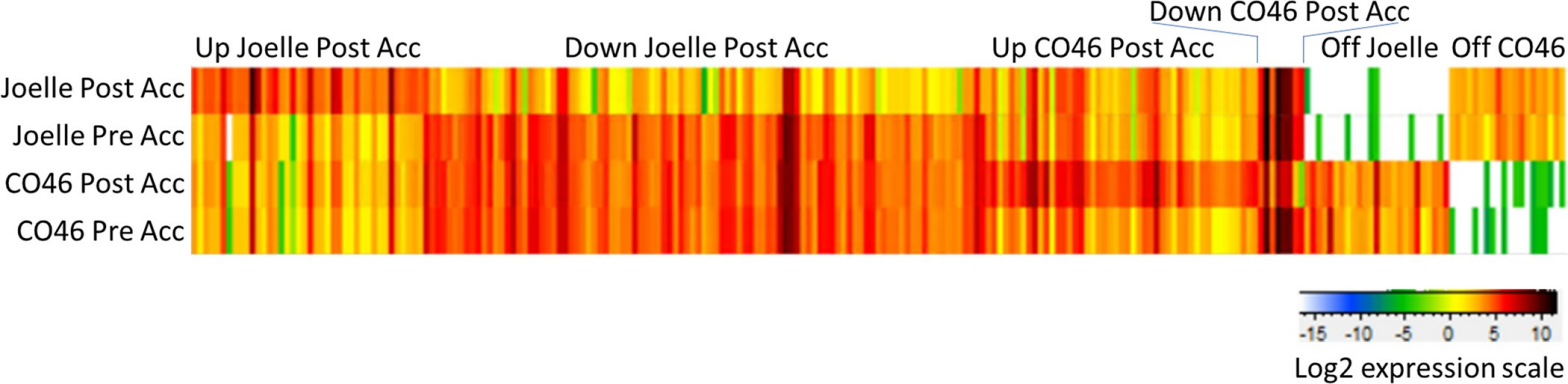
Coordinately regulated gene clusters. Heat map showing clusters of genes uniquely expressed in the various treatments. Treatments are labeled on the left for each row and Clusters are labeled above. The scale for gene expression is depicted as Log2 values of the mean expression from all three replicates of a given treatment is shown in the lower right.

One hypothesis for differences observed between the winter biotype Joelle and freezing sensitive summer biotype CO46 is that the cold-sensing or transduction signaling mechanism of CO46 does not respond appropriately to cold acclimating conditions. We hypothesized that a subset of these genes are likely to be important in the differential response of the summer and winter biotypes to freezing conditions. Of the 961 genes that were differentially expressed between the winter and summer biotypes following cold acclimation, there were 229 genes that were clustered into 6 groups (Fig 6) by the following criteria: 1) significant differential gene expression was observed in one biotype but not the other; 2) the gene also had significant differential expression between Joelle and CO46 after both biotypes were fully acclimated; 3) the gene was unchanged (Log2 <|0.75|) by the acclimation treatment in the other biotype; and/or 4) the gene was essentially off (FPKM <0.05) in one biotype or the other, both before and after cold acclimation (S1 Workbook). Twenty-three of these genes were essentially off in Joelle but expressed in CO46, and 20 were off in the freezing sensitive line CO46 but expressed in the freezing tolerant line Joelle (S1 Workbook). Two genes that were poorly expressed in CO46 under both control and cold-acclimating conditions were strongly up-regulated by cold in Joelle (S1 Workbook). Of the 229 genes clustered into specific groups (Fig 6), 38 were significantly up-regulated specifically in Joelle following cold treatment but had minimal change between non-cold-treated and cold-treated CO46. Likewise, 95 of these genes were down-regulated specifically in Joelle. Similarly, there were only 8 genes that were uniquely up-regulated and 45 genes that were uniquely down-regulated in CO46 when comparing non-cold-treated plants to those that were cold-treated (acclimated). Among genes down-regulated specifically in Joelle, there were several hormone-related transcription factor genes including one with similarity to *HOMEOBOX PROTEIN 6* and one with similarity to a *WRKY70*; involved in ABA and SA signaling respectively. Likewise, there were four genes associated with auxin signaling including two *INDOLE-3-ACETIC ACID INDUCIBLE 7* (*IAA7*) and one *INDOLE-3-ACETIC ACID INDUCIBLE 9* (*IAA9*), and an auxin catabolism encoding gene *GH3-like5*, that were down-regulated specifically in the winter biotype Joelle.

### Promoter analyses of coordinately-expressed gene clusters

**Fig 7:**
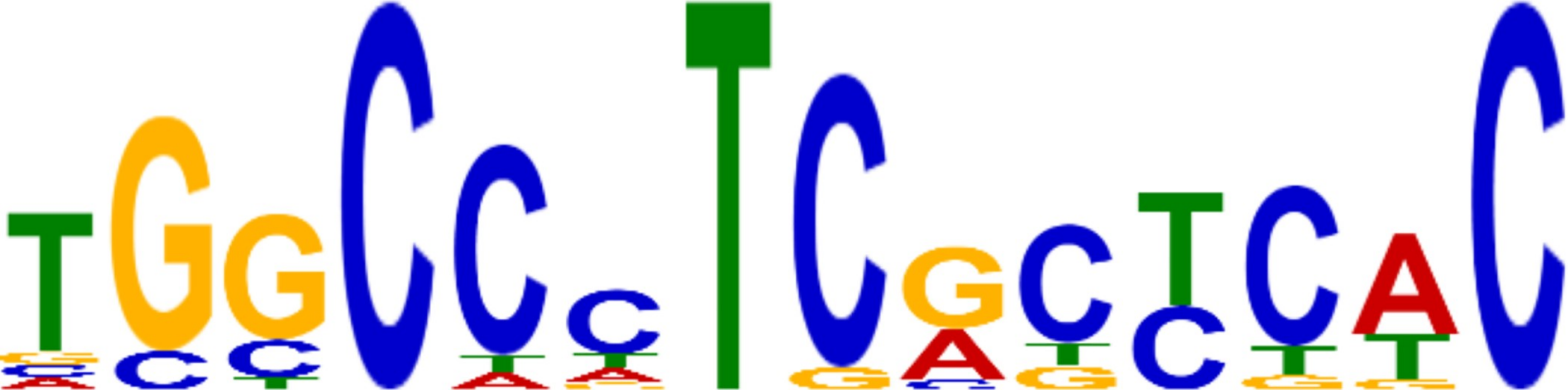
Over-represented promoter sequences. Results of MEME output showing consensus sequence. The size of the font for a given nucleotide is indicative of its representation in the consensus sequence among the 95 genes down-regulated in Joelle after cold acclimation.

Using the program MEME, we identified only one over-represented sequence of interest among the coordinately-regulated genes. This sequence was identified as over-represented among genes that were uniquely down-regulated (p value of 1.1e-003) in the winter biotype Joelle after cold treatment (Fig 7). Further examination of this sequence revealed that it was over-represented in Arabidopsis genes that have the gene ontology of “chloroplast.” Several other significantly over-represented motifs were also observed; however, they were generally stretches of mono- or di-nucleotides of insufficient complexity to justify further examination.

## Discussion

In this study, cold-acclimation increased survival of the winter annual biotype of Camelina (Joelle) compared with the summer annual biotype (CO46) after response to low temperature treatments (freezing)Camelina. Because we did not perform experiments to examine mechanisms of freezing damage (such as seeding plants with ice cystals to reduce super-cooling), we only focus on the underlying processes indicated by differences in gene expression. Because nearly all F_1_ progeny from 8 different crosses (including reciprocal crosses) survived freezing with minimal to no damage, our results suggest that survival after low temperature treatments is a dominant trait and not maternally inherited. An analysis of F_2_ progeny indicated the resulting phenotypes could most easily be explained if there were two dominant genes regulating survival after low temperature treatments. This can be inferred since phenotypes intermediate between the two parental biotypes were present at approximately a 9:6:1 ratio (no damage:damaged:dead). Although a single dominant gene could not be ruled out (Chi square p value was slightly greater than 0.05), the number of plants with an intermediate phenotype was too small to support this hypothesis. However, we cannot rule out more complex inheritance of the traits controlling survival after low temperature treatments.

To our knowledge, this is the first report linking genetic analyses to Camelina survival following a freezing-treatment. However, in other *Brassica* species, there have been several studies that mapped loci imparting differences between cultivars with divergent abilities to withstand freezing after acclimation [14]. However, there have been no successful map-based cloning of genes involved in freezing-treatment survival in any *Brassica* species that we know of. The closest has been the mapping of fairly large QTLs with several hundred variant genes in a recombinant inbreed population of Arabidopsis resulting from a cross between two lines- one collected from Sweden and one from Italy [15]. Differences in CBF sequences and activity were later associated with this diversity in survival after low temperature treatments [16].

### CBF regulon does not explain differences in the freezing-treatment survival of summer and winter biotypes

Induction of the CBF regulon has been reported to enhance freezing survival in several *Brassica* species [6,17]. Interestingly, in this study, an examination of the genes that were differentially expressed between pre- and post-cold acclimation indicated that the winter biotype Joelle and summer biotype CO46 both appeared to activate the CBF regulon. For example, the *CBF1* and *CBF2* genes were observed to be differentially expressed between pre- and post-cold acclimation in both biotypes. For example, *CBF1* shows over a 9 fold increase in expression in both CO46 and Joelle following acclimation. Also, many of the classic *COR* genes were induced in both biotypes. Also, few canonical *COR* genes were observed in the sets of genes differentially expressed uniquely in one biotype or the other. However, it should be noted that there were differences in the level of expression for a few *CBF* and *COR* genes with lower accumulation being observed in the freezing sensitive biotype CO46. Both *CBF2* and *CBF3* transcripts levels were 3–4 fold greater in Joelle than in CO46. However, although the CBF regulon may be impacted by the genetic differences observed between Joelle and CO46, this regulon does not appear to be the primary reason why summer and winter biotypes of Camelina differ in their ability to survive freezing temperatures. However, because we examined the transcriptome after 8 weeks under cold acclimating conditions, it is possible that transient differences in *CBF* expression or expression of *COR* genes responsive to CBFs may have been missed. Thus, although both biotypes induced *CBF*s by the end of the acclimation period, we can not rule out the possibility that the sensitive biotype did not initiate *CBF* transcription early enough to accumulate sufficient CBF protein for full acclimation. Also, we can not rule out post transcriptional modifcations which might impact the functionality of these *COR* gene regulators. Although any major post-transcrptional changes to CBFs might be expected to have greatly altered the expression of *COR* genes, we did not observe such differences between the two biotypes.

As observed for the two biotypes in this study, various other species appear to have active CBF regulons that are cold-induced and also appear to be inadequate for significantly improving freezing-treatment survival. For example, soybean (*Glycine max*) [9], potato (*Solanum ssp*), [18] and tomato (*Solanum lycopersicum*) [19] have active CBF pathways, yet are not all capable of significantly increasing freezing-treatment survival following cold-acclimation This does not always appear to result from inactive CBF genes as expression of the soybean or tomato CBF genes in Arabidopsis indicates they are functional [8,9]. Likewise, over-expression of the Arabidopsis CBF in freezing-sensitive soybeans induces a subset of COR genes in the transgenic plants, yet, they are generally not significantly more capable of surviving freezing than the non-transgenic controls [9]. These observations suggest that other factor(s) are required for either full functionality of the CBF regulon, or that there are other components besides the CBF regulon that are needed for increasing freezing survival following cold-acclimation. Thus, winter and summer biotypes of Camelina provide a unique opportunity to investigate the gene or genes that are responsible for the significant difference in freezing survival within the same species. For example, the results of this study provide some clues as to the mechanisms that distinguish between the cold-acclimation-induced increase in survival of Joelle compared with the decreased survival of CO46 following a freezing-treatment.

### Down-regulation of photosynthetic and light signaling processes correlate with survival after freezing

Down-regulation of photosynthetic processes has been associated with survival after freezing in multiple species [19]. Promoter regions of differentially expressed genes between Joelle and CO46 did not indicate differences among the well characterized transcription factors previously associated with cold-acclimation processes. However, the sequence TGGCCCTCGCTCAC was over-represented among genes that were down-regulated in Joelle. This same sequence is over-represented in genes with the ontology of “chloroplast” in Arabidopsis and may be the binding site defined as Sequences Over-Represented in Light-Induced Promoters (SORLIPs) [20]. Activation of photosynthetic processes under cold-induced metabolism could increase cellular levels of reactive oxygen species, likely leading to significant cellular damage [19]. Indeed, there are several lines of evidence suggesting a major factor in cold-acclimation processes involves protecting the photosynthetic apparatus from damage during low-temperature light exposure [19]. These observations suggest that positive activating factors binding this sequence are less functional or present in reduced levels in Joelle relative to freezing sensitive CO46.

Based on the above paradigm, we hypothesized that reduced oxidative stress would be observed in the winter biotype Joelle and increased in the freezing sensitive line CO46. In support of this hypothesis, the GO Term Enrichment tool (https://www.Arabidopsis.org/tools/go_term_enrichment.jsp) highlighted genes involved in stress and defense responses are over-represented among the 45 genes (Fig 3) noted as being specifically up-regulated by cold acclimation in the freezing sensitive CO46 (S3 Table). Likewise, similar over-represented gene ontologies are noted among the 95 genes that are specifically down-regulated only in Joelle, indicating that Joelle has undergone modifications that seem to have reduced the oxidative stress during cold-acclimating conditions. Numerous plant thioredoxins, a superoxide dismutase, a glutathione S transferase, a peroxidase, several Cinnamyl alcohol dehydrogenases are all preferentially expressed in CO46 while similar genes are down-regulated in the winter biotype Joelle (S1 Workbook).

In addition to light stress responses, we also observed changes in transcription factors associated with light-regulated gene expression (specifically long-day photoperiodism and flowering). For example, one transcription factor, encoding a nuclear factor Y, subunit B2, was specifically down-regulated in Joelle, whereas a gene encoding a transcription factor similar to *CONSTANS-like 2* was found to be up-regulated specifically in CO46 following cold acclimation. *CONSTANS-like 2* is associated with circadian responses in Arabidopsis [21]. Interestingly, *CONSTANS-like 2* has been observed to be generally up-regulated in Arabidopsis following cold-acclimation [22]. The fact that it is not differentially expressed in Joelle is intriguing. Other notable genes involved in light regulated signaling included a *EIN3-BINDING F BOX PROTEIN 2* previously associated with freezing tolerance [23], and which may regulate *PIF3* [24]. This gene was up-regulated in Joelle by cold acclimation but not in CO46 in response to cold. It is intriguing that EBF2 was specifically up-regulated in Joelle, since EBF2 regulates degradation of PIF3, which negatively regulates *CBF* expression [23]. Indeed, all these genes may impact the gating of CBF in response to diurnal signals noted by Fowler et al. (2005).[25].

### Genes associated with phytohormone responses and freezing survival

Among the genes that were essentially off in one biotype or the other, the only transcription factor represented had similarity to Arabidopsis MYB47 (most similar to a salt- and jasmonic acid (JA)-induced, AT1G18710). However, in Arabidopsis, this gene is not induced in response to cold (TAIR site) suggesting that it may serve a different function in Camelina. The observed down-regulation of a set of genes encoding proteins with high similarity to WKRY70 in this study is consistent with the depression of defense responses in the winter biotype Joelle. WRKY70 proteins act as negative regulators of the SA response pathways by reducing expression of genes involved in SA metabolism [26], and also act to integrate JA signals involved in defense of necrotrophic fungi [27].

ABA has also long been associated with stress responses [28]. The ABA regulatory transcription factor ABI1 that regulates *HOMEOBOX 6* [29] is down-regulated in Joelle, and HOMEOBOX6 has been reported to be a probable nexus of another CBF-independent cold-responsive regulon [30]. Because Joelle survives freezing better than CO46, this result is surprising, and suggests that down-regulation of *HOMEOBOX 6* is associated with freezing survival in our study. Both ABA and cold stress are also intimately associated with water relations [31]. Thus, the observation that a SHAKER-family gated outwardly-rectifying K+ channel [most similar to AT5G37500 and is regulated by cold, dehydration, abscisic acid (ABA), and JA] is up-regulated by cold in Joelle, but is essentially off in CO46 is also of potential interest.

Two other transcriptional regulators AUX/IAA7 and AUX/IAA9 are also down-regulated in the winter biotype Joelle. Similar associations of down-regulated auxin responses during cold acclimation have been observed in Arabidopsis [32]. These AUX/IAA proteins generally negatively regulate auxin signaling by binding to AUXIN RESPONSIVE FACTORS (ARFs) and preventing their interaction with auxin responsive elements in the promoters of auxin-regulated genes [33]. AUX/IAA9 has been implicated in regulation by the oxidative stress response through the action of TGACG SEQUENCE-SPECIFIC BINDING PROTEIN 2 (TGA2) [34]. This is of additional interest since the thioredoxin genes up-regulated in CO46 may also play a role in activating JA and SA regulated TGAs [35,36]. These results all point to oxidative stress as an important factor differentiating the winter biotype Joelle and CO46 in response to cold acclimation.

## Conclusion

Outcomes of this study indicate that the ability of summer and winter biotypes of Camelina to survive freezing conditions appears to involve genetic inheritance of one or two dominant genes. Transcriptome analysis highlighted a small number of genes that were differentially expressed between the two biotypes following cold acclimation, and which were uniquely expressed in one line or the other and/or were essentially off in one of the two biotypes. This set of genes highlighted hormone, photosynthetic and light signaling responses among genes down-regulated in the winter biotype Joelle. Likewise, the promoters from this set of specifically down-regulated genes were enriched in a sequence that is over-represented among Arabidopsis genes with the ontology “chloroplasts.” Several transcription factors and other regulatory genes were also highlighted including a gene with similarity to *MYB47*. Any or all of these genes could be involved in processes needed for freezing survival and/or associated with regulation of genes involved with oxidative stress responses. Further studies will be needed to determine if loss of function of any of these candidate genes significantly alters the freezing survival of Camelina. However, these studies highlight the importance of Camelina as a model system for identifying non-CBF-related differences that contribute to freezing survival and cold-acclimation processes in Brassicaceae.

## Supporting information

S1 Table Excel spreadsheet indicating damage scores from five F1 individuals each from several different recipricol crosses between Joelle and CO46. The highlighted cross was used to generate the F2 population that was advanced to the F5 recombinat inbred population phenotyped in this study.
(XLSX)

S2 Table Excel spreadsheet showing annotations, false discovery statistics for pairwise comparisons between treatments, and expression data for all annotated transcripts in the reference genome of Camelina. (XLSX)

S3 Table Excel spreadsheet showing results from PANTHER gene set enrichment analysis of genes up-regulated in CO46 following cold acclimation treatments. (XLSX)

S1 Workbook Excel workbook showing annotations, false discovery statistics for pairwise comparisons between treatments, and expression data for genes depicted in Fig 3. Each sub-cluster is noted on a separate page of the workbook.
(XLSX)
